# Prospective postmortem evaluation of 735 consecutive SARS-CoV-2-associated death cases

**DOI:** 10.1038/s41598-021-98499-3

**Published:** 2021-09-29

**Authors:** Antonia Fitzek, Julia Schädler, Eric Dietz, Alexandra Ron, Moritz Gerling, Anna L. Kammal, Larissa Lohner, Carla Falck, Dustin Möbius, Hanna Goebels, Anna-Lina Gerberding, Ann Sophie Schröder, Jan-Peter Sperhake, Anke Klein, Daniela Fröb, Herbert Mushumba, Sandra Wilmes, Sven Anders, Inga Kniep, Fabian Heinrich, Felicia Langenwalder, Kira Meißner, Philine Lange, Antonia Zapf, Klaus Püschel, Axel Heinemann, Markus Glatzel, Jakob Matschke, Martin Aepfelbacher, Marc Lütgehetmann, Stefan Steurer, Christoph Thorns, Carolin Edler, Benjamin Ondruschka

**Affiliations:** 1 Institute of Legal Medicine, University Medical Center Hamburg-Eppendorf, Hamburg, Germany; 2 Institute of Neuropathology, University Medical Center Hamburg-Eppendorf, Hamburg, Germany; 3 Institute of Medical Microbiology, Virology, and Hygiene, University Medical Center Hamburg-Eppendorf, Hamburg, Germany; 4 Institute of Pathology, University Medical Center Hamburg-Eppendorf, Hamburg, Germany; 5 Institute of Pathology, Marienkrankenhaus, Hamburg, Germany; 6 Department of Medical Biometry and Epidemiology, University Medical Center Hamburg-Eppendorf, Hamburg, Germany

**Keywords:** Infectious diseases, Epidemiology, SARS-CoV-2

## Abstract

Coronavirus disease 19 (COVID-19), caused by severe acute respiratory syndrome coronavirus 2 (SARS-CoV-2), has become a global pandemic with significant mortality. Accurate information on the specific circumstances of death and whether patients died from or with SARS-CoV-2 is scarce. To distinguish COVID-19 from non-COVID-19 deaths, we performed a systematic review of 735 SARS-CoV-2-associated deaths in Hamburg, Germany, from March to December 2020, using conventional autopsy, ultrasound-guided minimally invasive autopsy, postmortem computed tomography and medical records. Statistical analyses including multiple logistic regression were used to compare both cohorts. 84.1% (n = 618) were classified as COVID-19 deaths, 6.4% (n = 47) as non-COVID-19 deaths, 9.5% (n = 70) remained unclear. Median age of COVID-19 deaths was 83.0 years, 54.4% were male. In the autopsy group (n = 283), the majority died of pneumonia and/or diffuse alveolar damage (73.6%; n = 187). Thromboses were found in 39.2% (n = 62/158 cases), pulmonary embolism in 22.1% (n = 56/253 cases). In 2020, annual mortality in Hamburg was about 5.5% higher than in the previous 20 years, of which 3.4% (n = 618) represented COVID-19 deaths. Our study highlights the need for mortality surveillance and postmortem examinations. The vast majority of individuals who died directly from SARS-CoV-2 infection were of advanced age and had multiple comorbidities.

## Introduction

Severe acute respiratory distress syndrome-associated coronavirus-2 (SARS-CoV-2), the causative agent of coronavirus disease 2019 (COVID-19), was first identified in December 2019 in Wuhan, Hubei Province, China^1^ and was declared a pandemic by the World Health Organization (WHO) in March 2020^2^. As of May 18th, 2021, the outbreak of SARS-CoV-2 has spread to all continents, with about 164 million confirmed cases and over 3.4 million fatalities worldwide after contracting the respiratory virus^3,4^.

Internationally, the COVID-19 pandemic demonstrates that recording of total mortality in a dynamic infection event represents a particular challenge for harmonization and comparability of infection and case fatality figures. For this purpose, a systematic SARS-CoV-2 mortality monitoring has been established at the Institute of Legal Medicine (ILM) of the University Medical Center Hamburg-Eppendorf, Germany (UKE) in March 2020. At that time, there was little knowledge about the causes of death in such fatalities and the question was raised whether the patients died from or with SARS-CoV-2.

Considering the possible effects of SARS-CoV-2 on various organs, detailed knowledge of the organotropism of the virus, the identification of risk factors and the underlying ultimate causes of death were of particularly high clinical relevance^5–8^. Therefore, systematic investigations of SARS-CoV-2 associated deaths, defined as fatalities associated with a positive SARS-CoV-2 PCR test, deemed necessary to provide evidence for epidemiological clusters and patient cohorts underlying particular hazards for fatal courses of the disease.

The aim of this systematic postmortem evaluation was to classify all known SARS-CoV-2 associated deaths in the city of Hamburg, Germany, as COVID-19 or as non-COVID-19 deaths and to compare both groups, with respect to demographic, anthropometric and medical characteristics.

## Results

### Overall characteristic of the study

A total of 735 SARS-CoV-2 associated deaths were analyzed. Conventional autopsies were performed in 38.5% (n = 283). 5.6% (n = 41) of deaths were investigated by usMIA. In total, 55.9% (n = 411) received a PMCT and 35.9% (n = 264) were classified by medical record review only. In 34.6% (n = 254) several examinations were performed consecutively. CA was able to assign a definite cause of death in 99.3% of cases (281/283), followed by usMIA at 90.2% (37/41), isolated PMCT at about 87.9% (138/157) and medical record alone at about 83.0% (219/264) (see Supplemental Table S1).

A total of 618 cases (84.1%) were classified as COVID-19-related, including 254 (41.1%) by CA. The remaining 15.9% (n = 117) were divided into non-COVID-19 deaths and unclear causes of death (9.5%; n = 70). Unclear cause of death was the most frequent (64.3%) in cases limited to medical record review only due to insufficient data and/or missing consent to postmortem examination.

Overall (COVID-19 and non-COVID-19 death in total), 6.2% (n = 41) of patients died at home and 24.4% (n = 162) in nursing homes. The majority of 68.4% (n = 445) died in hospital, of which 38.9% (n = 259) died on the normal ward, 29.2% (n = 194) in the intensive care unit and 0.3% (n = 2) in the emergency room. In 7 cases, there was no or unclear information. The overall proportion of nursing home residents of all deaths (N = 735) investigated was 52.9% (n = 389). Regarding the survival time of the whole collective, we found a substantially longer time interval of first positively confirmed swab PCR test until date of death of hospitalized patients from May 2020 on (p = 0.011; median 6 days [March–April] versus 11 days [May–December]), but no comparable increase in outpatients (p = 0.328; median 8 days for both periods). Tables 1 and 2 show the distribution of the reported cases within the defined pandemic waves.

**Table 1:** Baseline characteristics of COVID-19 deaths and non-COVID-19 deaths (total N = 665; all unclear cases excluded).

	COVID-19 death				Non-COVID-19 death	
	1st wave	2nd wave	p value	Total		p value
	(n = 235)	(n = 383)		(n = 618)	(n = 47)	
**Sex**			0.645			0.199
Male^a^	125 (53.2%)	211 (55.1%)		336 (54.4%)	21 (44.7%)	
Female^a^	110 (46.8%)	172 (44.9%)		282 (45.6%)	26 (55.3%)	
**Age, years**			0.117			0.456
Median^b^	82.0 (31.0–99.0)	83.0 (29.0–100.0)		83.0 (29.0–100.0)	84.0 (36.0–102.0)	
IQR	75.0–87.0	77.0–89.0		76.0–88.0	78.0–90.0	
Mean^c^	80.0 (10.8)	81.3 (10.6)		80.8 (10.6)	81.8 (11.7)	
95% CI	75.0–87.0	80.3–82.4		80.0–81.8	78.4–85.3	
**Male, years**			0.041*			0.515
Median^b^	80.0 (31.0–99.0)	82.0 (46.0–99.0)		81 (31.0–99.0)	81.0 (56.0–91.0)	
IQR	71.5–86.0	76.0–88.0		74.0–87.0	73.0–84.5	
Mean^c^	77.1 (11.4)	80.4 (10.3)		79.4 (10.8)	87.2 (9.5)	
95% CI	75.8–79.9	79.0–81.8		87.3–80.6	73.9–82.6	
**Female, years**			0.750			0.128
Median^b^	84.0 (49.0–99.0)	84.0 (29.0–100.0)		84.0 (29.0–100.0)	87.0 (36.0–102.0)	
IQR	78.0–89.0	78.0–90.0		78.0–89.0	78.8–92.0	
Mean^c^	82.4 (9.4)	82.4 (10.8)		82.4 (10.3)	84.7 (12.7)	
95% CI	80.6–84.2	80.8–84.0		81.2–83.6	79.6–89.2	

This table displays patient characteristics and demographics of COVID-19 and non-COVID-19 deaths.

*IQR* interquartile range, *CI* confidence interval.

^a^Number (%), ^b^median (range), ^c^mean (standard deviation). Statistically significant p values are highlighted in bold (*p < 0.05), the remaining values stayed non-significant.

**Table 2:** Place of death of COVID-19 deaths and non-COVID-19 deaths (total N = 665; all unclear cases excluded).

	COVID-19 death				Non-COVID-19 death	
	1st wave	2nd wave	p value	Total		p value
	(n = 235)	(n = 383)		(n = 618)	(n = 47)	
**Place of death**			0.034*			< 0.001**
Outpatient^a^	62 (26.4%)	114 (29.8%)		176 (28.5%)	28 (59.6%)	
At home	13 (5.5%)	22 (5.7%)		35 (5.7%)	6 (12.7%)	
Retirement	49 (20.9%)	92 (24.2%)		141 (22.8%)	21 (44.7%)	
Other	–	–		–	1 (2.1%)	
Hospital^a^	169 (71.9%)	267 (69.7%)		436 (70.6%)	19 (40.4%)	
ICU	85 (36.2%)	105 (27.4%)		190 (30.7%)	4 (8.5%)	
Normal ward	82 (34.9%)	162 (42.3%)		244 (39.5%)	15 (31.9%)	
Emergency department	2 (0.9%)	–		2 (0.3%)	–	
No information^a^	4 (1.7%)	2 (0.5%)		6 (1.0%)	–	
**Place of death of retirement residents**			0.006*			0.005*
Proportion of retirement residents^a^	103 (43.8%)	219 (57.2%)		322 (52.1%)	26 (55.3%)	
Retirement	47 (45.6%)	90 (41.1%)		137 (42.5%)	20 (76.9%)	
Hospital	53 (51.5%)	127 (58.0%)		180 (55.9%)	6 (23.1%)	
	25 (24.3%)	34 (15.5%)		59 (18.3%)	–	
	27 (26.2%)	93 (42.5%)		120 (37.3%)	6 (23.1%)	
	1 (1.0%)	–		1 (0.3%)	–	
No information	3 (2.9%)	2 (0.9%)		5 (1.6%)		

This table displays place of death of COVID-19 and non-COVID-19 deaths. In addition, the place of death of home residents is displayed.

*ICU* intensive care unit.

^a^Number (%). Statistically significant p values are highlighted in bold (*p < 0.05; **p < 0.001), the remaining values stayed non-significant.

### COVID-19 deaths

54.4% (n = 336) of the COVID-19 death group were men. The median age was 83.0 years (IQR 76.0–88.0) with a higher age of women compared to men (median 84.0 [IQR 78.0–89.0] versus 81 [IQR 74.0–87.0] years), see Table 1. Only seven deceased were younger than 50 years.

28.5% (n = 176) died in the outpatient and 70.6% (n = 436) in the hospital setting, with more patients died on normal ward (n = 244; 39.5%) than in the ICU (n = 190; 30.7%).

82.8% (n = 322) nursing home residents were defined as COVID-19 deaths representing 52.1% of all COVID-19 deaths (n = 618) in our cohort (Table 2). Supplemental Table S2 shows the results of the multiple logistic regression of the COVID-19 death group.

### Non-COVID-19 death

47 cases (6.4%; 44.7% men) were defined as non-COVID-19 deaths (Table 1). Deceased men were younger with a median age of 81.0 years (IQR 73.0–84.5) than women at a median age of 87.0 years (IQR 78.8–92.0). Most of the deceased with an alternating cause of death died in the outpatient setting (59.6% versus 28.8%, p < 0.001; Table 1). Tables 1 and 2 lists patients’ characteristics and demographics factors.

### Autopsy cohort

In total 283 CA were performed. Table 3 shows demographic factors and place of death in detail for the autopsy group. Of the 254 COVID-19 deaths in the autopsy group, most patients died of pneumonia and/or diffuse alveolar damage (73.6%; n = 187), whereas cardiac associated fatalities were strongly represented in the non-COVID-19 group (70.4%, n = 19), see Table 4.

**Table 3:** Baseline characteristics of the autopsy cohort.

	COVID-19 death	Non-COVID-19 death	p value
	(n = 254)	(n = 27)	
**Sex**			0.921
Male^a^	139 (54.7%)	14 (51.9%)	
Female^a^	115 (45.3%)	13 (48.2%)	
**Age, years**			0.643
Median^b^	82.0 (29.0–100.0)	83.0 (36.0–96.0)	
IQR	75.0–87.0	73.0–90.0	
Mean^c^	79.4 (12.0)	79.7 (13.5)	
95% CI	77.9–80.4	74.4–85.0	
**Male**			0.683
Median^b^	80.0 (31.0–99.0)	78.5 (56.0–91.0)	
IQR	71.0–85.0	73.0–84.0	
Mean^c^	77.2 (12.0)	76.4 (11.0)	
95% CI	75.2–79.2	70.0–82.7	
**Female**			0.262
Median^b^	85.0 (29.0–100.0)	86.0 (36.0–96.0)	
IQR	77.0–89.0	82.0–92.0	
Mean^c^	82.0 (11.4)	83.3 (15.3)	
95% CI	79.9–84.1	74.0–92.6	
**Below 50 years**			
Number^a^	6 (2.4%)	1 (3.7%)	
Male^a^	4 (66.7%)	–	
Female^a^	2 (33.3%)	1 (100.0%)	
**Place of death**			0.001*
Outpatient^a^	87 (34.3%)	18 (66.7%)	
At home	21 (8.3%)	5 (18.5%)	
Retirement	66 (26.0%)	12 (44.4%)	
Other	–	1 (3.7%)	
Hospital^a^	166 (65.4%)	9 (33.3%)	
Normal ward	100 (39.4%)	7 (25.9%)	
ICU	64 (25.2%)	2 (7.4%)	
Emergency department	2 (0.8%)		
No information^a^	1 (0.4%)		

This table displays patient characteristics and place of death of COVID-19 and non-COVID-19 deaths of the autopsy cohort.

*ICU* intensive care unit, *CI* confidence interval.

^a^Number (%), ^b^median (range), ^c^mean (standard deviation). Statistically significant p values are highlighted in bold (*p < 0.05), the remaining values stayed non-significant.

**Table 4:** Comorbidities of the autopsy cohort.

	**COVID-19 death**	**Non-COVID-19 death**	**p value**
	(n = 254)	(n = 27)	
**Comorbidities**^**#**^			0.689
Cardiovascular^a^_,_^d^	226 (89.0%)	25 (92.6%)	
Pulmonary^a^_,_^d^	124 (48.8%)	18 (66.7%)	
Neurological^a^_,_^d^	119 (47.0%)	7 (25.9%)	
Renal^a^_,_^d^	94 (37.0%)	9 (33.3%)	
Endocrine^a^_,_^d^	71 (28.6%)	7 (25.9%)	
Oncologic^a^_,_^d^	53 (20.9%)	3 (11.1%)	
Liver a^a^_,_^d^	17 (6.7%)	1 (3.7%)	
Pancreatic^a^_,_^d^	4 (1.6%)	–	
Immunological^a^_,_^d^	12 (4.7%)	–	
Psychological^a^_,_^d^	5 (2.0%)	–	
Chronic inflammation^a^_,_^d^	4 (1.6%)	–	
Other^a^_,_^d^	16 (6.3%)	1 (3.7%)	
**Numbers of pre-existing condition**			
Average	2.9	2.6	
0^a^	3 (1.2%)	–	
1^a^	29 (11.5%)	3 (11.1%)	
2^a^	61 (24.1%)	12 (44.4%)	
3^a^	85 (33.6%)	7 (25.9%)	
4^a^	48 (19.0%)	3 (11.1%)	
5^a^	20 (7.9%)	1 (3.7%)	
6^a^	7 (2.8%)	1 (3.7%)	
**BMI, kg/m**^**2**^			0.194
Number^a^	204 (80.3%)	18 (66.7%)	
Median^b^	24.7 (12.4–53.3)	21.8 (11.4–43.6)	
IQR	20.4–28.8	19.3–25.5	
Mean^c^	25.5 (7.3)	23.1 (7.3)	
95% CI	24.5–26.5	19.5–26.7	
Underweight^a^	30 (14.7%)	3 (16.7%)	
Normal weight^a^	76 (37.3%)	9 (50.0%)	
Pre-obesity^a^	55 (26.7%)	4 (22.2%)	
Obesity class I^a^	22 (10.8%)	1 (5.6%)	
Obesity class II^a^	8 (3.9%)	1 (5.6%)	
Obesity class III^a^	13 (6.4%)	–	

This table displays comorbidities of COVID-19 and non-COVID-19 deaths of the autopsy cohort.

*BMI* body mass index, *IQR* interquartile range, *CI* confidence interval.

^#^Comorbidities were counted by macroscopic findings and available clinical data, ^a^number (%), ^b^median (range), ^c^mean (standard deviation), ^d^multiple inclusions of one patient in the various categories possible. p values stayed non-significant within this table.

Further, thromboembolic complications (9.4%, n = 24) and other organ failures led to death in the autopsy group. In 158 (62.2%) COVID-19 cases and in 11 (40.7%) non-COVID-19 cases detailed preparation of the lower extremities took place. In this subcohort, thromboses were found in 39.2% (n = 62) of 158 cases and likewise pulmonary embolisms were found in 22.1% (n = 56) of 253 cases (Table 5).

**Table 5:** Autopsy characteristics of the autopsy cohort.

	COVID-19 death	Non-COVID-19 death	p value
	(n = 254)	(n = 27)	
**PMI, days**			
Number^a^	245 (96.5%)	25 (92.6%)	
Median^b^	4.0 (0.0–36.0)	5.0 (1.0–24.0)	
IQR	2.0–7.0	3.0–9.5	
Mean^c^	5.4 (4.8)	7.1 (6.0)	
95% CI	4.8–6.0	4.6–9.5	
**Cause of death**^**a**^			< 0.001**
Pneumonia, DAD	187 (73.6%)	1 (3.7%)	
Thrombembolism	24 (9.4%)	–	
Cardial	15 (5.9%)	19 (70.4%)	
Inflammatory	13 (5.1%)	4 (14.8%)	
Organ dysfunction	5 (2.0%)	–	
Oncologic	2 (0.8%)	1 (3.7%)	
**Thrombosis**^**a**^			
Number^d^	158 (62.2%)	11 (40.7%)	
Present at autopsy	62 (39.2%)	0 (0.0%)	
**Embolism**^**a**^			
Number^e^	253 (99.6%)	27 (100.0%)	
Present at autopsy	56 (22.1%)	2 (7.4%)	
**Combined lung weight, g**			0.009*
Number^a^	225 (88.6%)	23 (85.2%)	
Median^b^	1425.0 (1110.0–1872.5)	1080.0 (815.0–1485.0)	
Mean^c^	1508.3 (533.5)	1213.9 (456.8)	
95% CI	1438.2–1578.4	1016.4–1411.4	

This table displays autopsy characteristics of COVID-19 and non-COVID-19 deaths of the autopsy cohort.

*PMI* postmortem interval, *CI* confidence interval, *DAD* diffuse alveolar damage.

^a^Number (%), ^b^median (range), ^c^mean (standard deviation), ^d^preparation of the lower extremities was performed in 158 COVID-19 and 11 non-COVID-19 cases, ^e^no information was available in one case. Statistically significant p values are highlighted in bold (*p < 0.05), the remaining values stayed non-significant.

Comorbidities were recorded from morphological findings at autopsy and available clinical data. The most common diagnoses were cardiovascular comorbidities with 89.0% (n = 226) (Table 4). Among these, there was a significantly increased probability that they also had a previous neurological disease (p = 0.015; Supplemental Table S2). On average, COVID-19 decedents had 2.9 preexisting conditions. The patients who died in hospital had more pre-existing conditions overall (p = 0.006).

The median BMI evaluated in 204 cases was 24.7 kg/m^2^ (IQR 20.4–28.8). In 50 CA cases BMI calculation was deferred because of severe edema or body weight or height was missing. 21.1% (n = 43) of the deceased were obese with no difference compared to non-COVID-19 deaths (p = 0.194), see Table 4. Combined lung weight was higher in COVID-19 deaths compared to the non-COVID-19 deaths, respectively (p = 0.009; median 1425.0 g [IQR 1110.0–1872.5] versus 1080.0 g [IQR 815.0–1485.0]), see Table 5.

Supplemental Table S2 shows the results of the multiple logistic regression in the CA collective of the COVID-19 death group.

### Comparison to official numbers showed minor deviations in total numbers but similar curve shapes and an excess mortality in 2020

**Figure 1:**
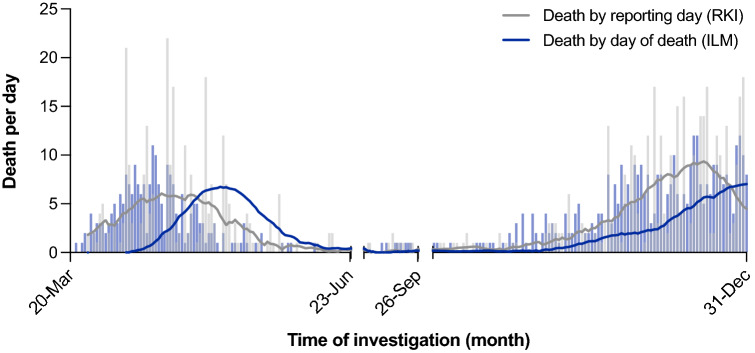
Reported SARS-CoV-2 associated deaths per day by the Robert Koch Institute (RKI)^9^ in grey scales versus the reported COVID-19 deaths per day by the Institute of Legal Medicine (ILM) Hamburg in blue scales.

In contrast to the ILM reported death cases by the date of death, the death cases reported by the Robert-Koch-Institute (RKI), the German government’s central scientific institution in the field of biomedicine, show a higher peak mostly at the beginning of the weeks depending on the official reports (Fig. 1). Overall, the 618 COVID-19 deaths reported to Hamburg’s health authority in 2020 resulted in a difference of 14 cases (618 vs. 632 cases) compared to the SARS-CoV-2-associated deaths in Hamburg reported by the RKI^9^.

**Figure 2:**
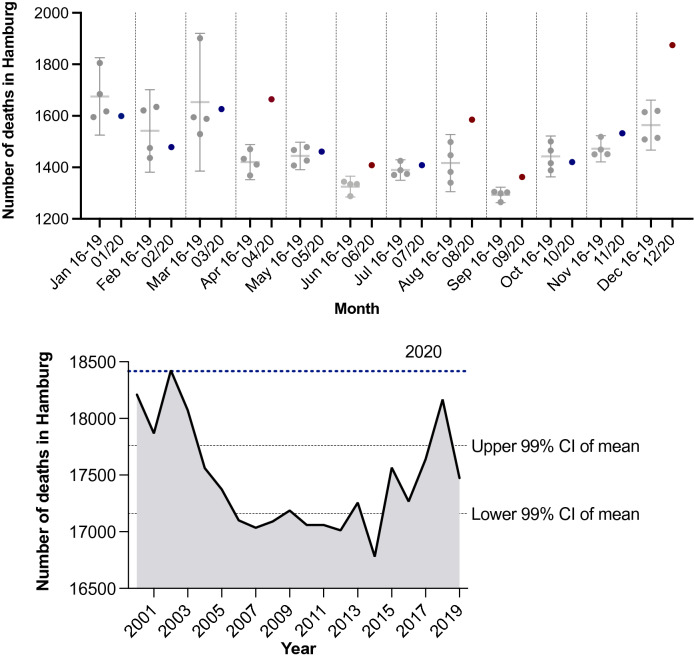
Number of deaths in general in Hamburg. Top: comparison of the monthly number of deaths in 2020 with the monthly average of 2016–2019, displayed with 95% confidence interval (CI) of the mean, blue dots for 2020 numbers within and red dots outside this CI. Bottom: comparison of the annual number of deaths between 2000 and 2019 with 99% CI of the mean as greyish dotted lines and the total number for 2020 with 18,417 fatalities in dotted blue line. Underlying data was available at the Federal Statistical Office Germany (DESTATIS)^11^.

Between 2000 and 2019, an average of 17,461 people died in Hamburg each year. Given the 18,417 deaths in 2020 in Hamburg^10,11^ this corresponds to an increase in deaths of about 5.5% for 2020 alone, of which 3.4% (n = 618) represent COVID-19 deaths (Fig. 2).

## Discussion

Our main results show that the majority of deaths associated with SARS-CoV-2 positivity died due to COVID-19 (84.1%), which is consistent with previous reports^7,12,13^. Most of the COVID-19 deaths were male with a high median age of 83.0 years. Our data confirm that the mortality rate is increased in patients over 60 years of age suffering from COVID-19^14,15^. In contrast, WHO and RKI figures on confirmed non-fatal COVID-19 cases show an increase in the 20–60 age group compared to the 60 + age group^4,9^. Patients under 60 years of age tend to have less severe symptoms and higher recovery rates than older patients^15^, which is consistent with our data on fatal cases decreasing with age (1% of the sample < 50 years)^15^. Epidemiologically, men have a higher risk of severe COVID-19 sequelae than women^16^. The number of confirmed positive cases is roughly equal in men and women^4,9,17^, but the male-dominated sex ratio in COVID-19 deaths has been confirmed in our study and worldwide^18,19^.

The deceased in our autopsy group suffered from many pre-existing conditions, especially of cardiovascular origin. The autopsy group of COVID-19 deaths had an average of 2.9 comorbidities. In contrast, Rommel et al.^20^ described fewer comorbidities, an average of 1.6 for the German population, figures that were related to reported deaths by the RKI.

However, these data were evaluated purely anamnestically; a post-mortem examination was not carried out. Previous studies have also reported that obesity is a relevant pre-existing condition^21–23^. Here, our results show a heterogeneous distribution between BMI values. Interestingly, only 21% of the COVID-19 death group were obese. This roughly corresponds to the national average for Germany, as about 22% of the German population had a BMI ≥ 30 in 2017 (most recent data)^24^. It should be noted that older patients in general, especially in our cohort of nursing home residents, are more prone to cachexia, malnutrition or underweight, which may also be an underestimated risk factor.

Our data confirm the above studies that age, gender and comorbidities are risk factors for fatal outcome^20^. Although women in the COVID-19 death group were older than men, there were no differences in sex, age or type of comorbidity when comparing the COVID-19 to the non-COVID-19 death group, highlighting the influence of underlying risk factors for fatal disease outcome in older patients. Notably, pre-existing neurological conditions were common in the hospitalized COVID-19 death group.

The main cause of death in our cohort and others was SARS-CoV-2 induced lung injury^18,25,26^. The virus infects airway epithelial cells^27^, leading to diffuse alveolar damage, edema and a marked increase in lung weight in our COVID-19 autopsy group, as previously reported^7,28^. It is noteworthy that the histological changes in the lungs were heterogeneously distributed, corresponding with our radiological findings of patchy dullness opacities, which most likely indicate a diffuse spread of the virus in the respiratory tract^28,29^. In addition, multi-site organ tropism has been reported to be favored in tissues with high expression of the angiotensin converting enzyme 2 receptor^6,30–36^. As venous thromboembolism was increasingly seen as a complication, since the beginning of May 2020, adapted anticoagulation has also been used in intensive care in Hamburg^7,28^. Although pulmonary emboli and deep vein thrombosis of the lower extremities were still diagnosed in some of the autopsies. The survival time of hospitalized patients increased from May 2020 onwards.

Notwithstanding the important role of pathology in clarifying the cause of death in clinical cases, our results show the high importance of forensic autopsy in the context of pandemics^26,37^. 28.5% of COVID-19 deaths died in the outpatient setting, which underlines the need for close cooperation between the disciplines involved. The RKI figures show a comparable distribution of places of death as in our cohort, with about 25% of deaths occurring outside the hospital^10,38^. It was notable that many cases in the non-COVID-19 death group occurred in the outpatient setting, mostly due to minor or non-specific symptoms of disease.

Interestingly, Hamburg had a slightly increased excess mortality rate of about 4.4% compared to 2016–2019 and 5.5% compared to the last two decades in 2020^9,10,39^.

Compared to Hamburg, excess mortality was also found nationwide, consistent with the number of people who died from or with SARS-CoV-2 by the end of September 2020^20^. About 3/4 of these higher numbers can be explained by the number of COVID-19 deaths in 2020^20^. Other indirect effects such as threshold increases for the utilization of outpatient treatment or hospitalizations due to other diseases may have additionally influenced the total number.

Reliable information on mortality is therefore of paramount importance to establish sound public health policies and to literally fight the pathogens of emerging infections. Previous and ongoing pandemics have shown that autopsy is a powerful tool to understand the underlying pathology of a disease^27,40,41^. The need for a standardized, nationwide recording method is illustrated by a difference of 14 cases between RKI and ILM of Hamburg deaths by the end of 2020 (632 vs. 618 cases)^9,10^.

Accurate postmortem diagnosis during the initial phase of an emerging epidemic represents an improvement in the identification of the specific etiological agent, which has significant implications for disease surveillance. Although our data show that CA is the most accurate way to assess the cause and manner of death, autopsy rates worldwide have declined significantly in recent decades and are below 5% in Germany^42–46^. Compared to this percentage, the autopsy rate of 38.5% reported in this study appears exceptionally high. Interestingly, the autopsy cohort did not have any variables that were statistically different from the overall cohort. It is therefore conceivable that due to the high autopsy rate in our study, the findings made for CA, e.g., on comorbidities and BMI, are transferable to the overall cohort of COVID-19 deaths.

To further increase the number of morphology-based postmortem examinations, usMIA was implemented as an alternative to CA. Other imaging modalities, including CT, MRI and/or robotic biopsy collection, can only be performed in centers of excellence and require significant budgets and infrastructure^47–49^. In contrast, usMIA is flexible, less expensive and has also been tested with promising results and own experience^50–55^. This methodology represents a research method that can be useful, especially in countries where mortality data are not available, to counteract the loss of numbers from CA in one's own setting^50–54,56,57^. Notwithstanding the advantages of minimally invasive autopsy, there are diagnostic limitations of this approach, particularly due to the accuracy in localizing pathological findings to ensure representative sampling. Further studies are needed to demonstrate concordance between CA and MIA and thus to verify the reliability of MIA.

## Limitations

Firstly, only part of the cohort was subjected to CA. A relevant part of the assessment was based only on the evaluation of medical records and available documents. Secondly, a shorter PMI (72 h) was assumed as a prerequisite for performing a CA or usMIA (procedural putrefactive gas inclusions with lower informative value by usMIA) during follow-up for organ samples and further laboratory approaches. Unfortunately, numerous cases did not fulfil this qualitative preselection because they were reported too late to the ILM or were not known. Thirdly, the diagnostic efficiency of a combined usMIA/PMCT as an evaluation method needs to be compared with CA in further studies. Finally, slight differences in individual assessment may have occurred due to interindividual perception and subjective interpretation of the morphological findings.

## Conclusion

Accurate information on the specific causes of death in patients dying from emerging infections in particular is scarce, which is why the question of whether patients died from or with SARS-CoV-2 has arisen since the start of the pandemic in 2020.

We show here that the vast majority died directly from SARS-CoV-2 infection as COVID-19 deaths, had advanced age and multiple comorbidities.

The multimodal "Hamburg Way", i.e., a systematic evaluation of all SARS-CoV-2-associated deaths in the city of Hamburg, only became possible through close cooperation with the Department of Social Affairs and Health, which led to an adjustment of the medical treatment of COVID-19 patients in the early phase of the pandemic. In addition, the systematic recording of non-clinical deaths led to a comprehensive population-based recording and evaluation of deaths. However, such an evaluation would not have been possible without the consent of the relatives to the scientific evaluation and the direct networking with the local health offices and special legal foundations in Hamburg.

## Materials and methods

### Study design, organizational structure and study cohort

All reported SARS-CoV-2-associated deaths (defined as ante- and/or post-mortem confirmed SARS-CoV-2 infection of a person at any point in time) of Hamburg citizens were evaluated at ILM Hamburg in collaboration with the health authorities in the period from mid-March 2020 to 30th September (first wave) and 1st October to 31st December 2020 (second wave).

**Figure 3:**
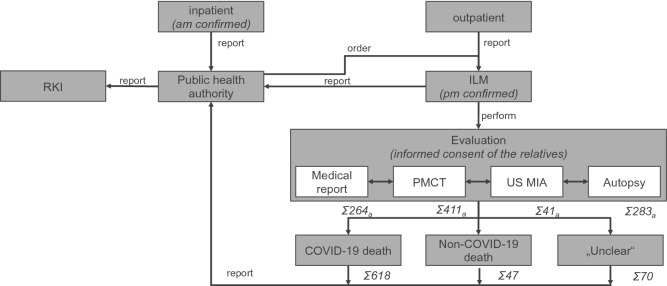
Flow chart of SARS-CoV-2 associated death evaluation at the ILM. This flow chart depicts processes and steps for the evaluation of 735 SARS-CoV-2 associated deaths in cooperation with the Hamburg public health authority. After the evaluation process based on medical report, postmortem computed tomography (PMCT), ultrasound-guided minimally invasive autopsy (usMIA) and conventional autopsy, 618 deaths were classified as COVID-19 deaths, 47 deaths were non-COVID-19 deaths and 70 deaths remained unclear. *am* antemortem, *pm* postmortem, *ILM* Institute of Legal Medicine, *RKI* Robert Koch Institute; a multiple inclusion of one patient in the various categories possible.

To scrutinize the reports and in order not to overlook any unknown cases, all deceased admitted to ILM were screened for viral SARS-CoV-2 RNA using a throat swab followed by immediate RT-qPCR at the Institute of Microbiology, Virology and Hygiene, UKE as previously described^58^. Figure 3 displays the practical process of reports, orders, investigations and process chains between local and national authorities involved.

Demographic (place of death, age, sex), and medical characteristics (cause of death) were collected for the overall collective. Further anthropometric (BMI) and medical/autopsy characteristics (survival time, comorbidities, combined lung weight, postmortem interval, thromboses and embolisms) were added for the autopsy sub-group.

Institutional review board approval from the independent ethics committee of the Hamburg Chamber of Physicians was obtained for this study (reference numbers 2020-10353-BO-ff and PV7311). The study complied with the tenets of the Declaration of Helsinki. Informed consent was obtained from a next of kin or legal representatives and authorities for the death case evaluation. All data were pseudonymized according to the guidelines from the central ethics commission of the federal medical association.

### Evaluation methods

Depending on the order and the consent of the relatives, a four-step concept was established to determine the underlying cause of death and thus categorize the individual cases into COVID-19 and non-COVID-19 deaths. The final categorization of each case was done by consensus with a supervisor's decision.

The evaluation was based on individual case decisions in descending order, dependent from the level of consent with

1. conventional autopsy (CA) through opening of all three body cavities.
2. ultrasound-guided minimally invasive autopsy through ultrasound-guided needle puncture of the organs (usMIA; LOGIQe 5417728-100, GE Medical Systems Ultrasound and Primary, USA).
3. postmortem computed tomography (PMCT) of the whole body (Philips Brilliance 16-slice multidetector scanner, Hamburg, Germany; full-body scan: slice thickness 1 mm; pitch, 1.5; 120 kV; 230–250 mA; in addition thorax scan with higher resolution) in accordance with an established protocol^48^.
4. an assessment of medical records, laboratory results, patient history and death certificate information to determine the most plausible cause of death.

Using the above-mentioned diagnostic tools, the cases were evaluated and classified into categories adapted to Edler et al.^8^.

Until autopsy, pmCT or usMIA the bodies were stored at 4 °C after death constantly. In case of short PMI (< 72 h) a standardized and extended tissue sampling was performed (see Supplemental Table S3, S4). Therefore, samples were fixed in buffered 4% formaldehyde for histopathological assessment or were made accessible for further laboratory examination methods by cryopreservation. In those cases with consent for further neuropathological examinations, the brain was examined at the Institute of Neuropathology of the UKE^30^ after being fixed in toto in 4% formaldehyde, as well.

For the medical record evaluation only, COVID-19 death was assessed, if COVID-19 has already been clinically assumed to be the cause of death and this has been substantiated by imaging, clinical investigations, typical symptoms and laboratory results.

On PMCT, evidence of peripheral or disseminated ground-glass opacities with bilateral ground dense nodules, areas of consolidations and crazy paving patterns led to classification as COVID-19 death^28^.

In usMIA, the combination of medical records, ultrasound and computed tomographic findings, as well as histology findings led to the classification of COVID-19 death. Typical findings on ultrasonography were consistent with general evidence of pneumonia, such as consolidations, enhanced B-lines (multifocal, confluent), an aerobronchogram and thickened pleural lines. In some cases, there were also subpleural indentations, as seen in peripheral pulmonary infarcts. Histological criteria of COVID-19 included diffuse alveolar damage (DAD), especially hyaline membranes, and activated pneumocytes, squamous metaplasia or organizing pneumonia.

At CA, a COVID-19 death was defined if the cause of death was found macroscopically within the pulmonary vasculature, in terms of embolism, or in the lungs as pneumonia and DAD. The cause of death was determined by postmortem examination, taking into account the medical history, if available, and any additional examinations in accordance with the guidelines of the German Society of Legal Medicine in its current version^59^. A high standard of diagnosis is ensured by many years of extensive experience in post-mortem and autopsy diagnosis. The diagnosis of a COVID-19 death required evidence of severe and fatal lung injury or other serious complications related to COVID-19 and excluded a competing cause of death, see Table 6.

**Table 6:** Categorization of COVID-19 deaths using a adapted categorization of Edler et al.^8^.

Category	Explanation
COVID-19 death	Autoptic pneumonia and/or ARDS and/or pulmonary embolism and/or infectious progress linked to COVID-19 as definite/probable/possible cause of death
Non-COVID-19 death	SARS-CoV-2 detection with cause of death not associated to COVID-19 (e.g. brain mass hemorrhage in hypertension, acute myocardial infarction in coronary thrombosis)
Unclear cases	The cause of death remains unknown, not evaluable

This table displays the established categorization of COVID-19 and non-COVID-19 death at the Institute of Legal Medicine, which was used to clarify all evaluated death cases corresponding to a RT-qPCR positive pre- or postmortem SARS-CoV-2 test.

*ARDS* acute respiratory distress syndrome.

Non-COVID-19 death were determined as acute and independently life-threatening conditions, such as pericardial tamponade.

If no definite lung changes were found by any of the examination procedures or data was inconclusive, and no other manifest cause of death was apparent, the cases were classified as “unclear”.

### Statistical analysis

This was an exploratory hypotheses-generating study. Therefore, no confirmatory analyses were conducted. The p values were therefore not adjusted for multiplicity and are used exclusively as descriptive measures. The assumption of a normal distribution was checked graphically using range (IQR) and compared by the Mann–Whitney U test. Categorical variables are summarized as counts and percentages and compared by the chi-square test or Fisher’s exact test, as appropriate.

Special attention was paid to the CA collective. In addition to above mentioned basic data, pre-existing medical conditions, body mass index (BMI) and autopsy findings were evaluated descriptively.

Furthermore, a multiple logistic regression analysis was performed with COVID-19 death versus non-COVID-19 death as dependent variable, separately for the whole collective and the CA collective. The independent variables were age, sex and place of death, and for the CA collective, additionally, pre-existing medical conditions and BMI.

Finally, the COVID-19 deaths in Hamburg identified by the ILM were correlated with the total number of SARS-CoV-2 infections in Hamburg and the number of deaths presented by the RKI^11^, as well as with the number of deaths in general in Hamburg in the past years (2000–2019) and per month (for the period 2016–2019)^10^.

Statistical analysis and graphical presentation of the results were done using the statistical software SAS (v9.4, SAS Institute Inc., Cary, NC, USA), GraphPad Prism® (v8.0, GraphPad Software Inc., La Jolla, USA), SPSS® (v10.0, SPSS Inc., New York, USA) and Microsoft Excel (version 16.16, Microsoft Corporation, Redmond, USA).

## Supplementary Information

Supplementary Information.

## Data Availability

The datasets generated and analyzed during the current study are available from the corresponding author on reasonable request.
