# Supplementary material for: Prospective postmortem evaluation of 735 consecutive SARS-CoV-2-associated death cases
Source: Sci Rep. 2021 Sep 29;11:19342. doi: 10.1038/s41598-021-98499-3 (PMC8481286; doi:10.1038/s41598-021-98499-3)
Supplement: Supplementary file 1 — Supplementary Information. [file 41598_2021_98499_MOESM1_ESM.docx]

Electronic supplementary material to:

Prospective postmortem evaluation of 735 consecutive SARS-CoV-2-associated death cases.

Antonia Fitzek¹*, Julia Schädler¹*, Eric Dietz¹*, Alexandra Ron¹*, Moritz Gerling¹, Anna L. Kammal¹, Larissa Lohner¹, Carla Falck¹, Dustin Möbius¹, Hanna Goebels¹, Anna-Lina Gerberding¹, Ann Sophie Schröder¹, Jan-Peter Sperhake¹, Anke Klein¹, Daniela Fröb¹, Herbert Mushumba¹, Sandra Wilmes¹, Sven Anders¹, Inga Kniep¹, Fabian Heinrich¹, Felicia Langenwalder¹, Kira Meißner¹, Philine Lange¹, Antonia Zapf^6^, Klaus Püschel¹, Axel Heinemann¹, Markus Glatzel², Jakob Matschke², Martin Aepfelbacher³, Marc Lütgehetmann³, Stefan Steurer^4^, Christoph Torns^5^, Carolin Edler¹**, Benjamin Ondruschka¹**

¹ Institute of Legal Medicine, University Medical Center Hamburg-Eppendorf, Hamburg, Germany

² Institute of Neuropathology, University Medical Center Hamburg-Eppendorf, Hamburg, Germany

³ Institute of Medical Microbiology, Virology, and Hygiene, University Medical Center, Hamburg-Eppendorf, Hamburg, Germany

^4^ Institute of Pathology, University Medical Center, Hamburg-Eppendorf, Hamburg, Germany

^5^ Institute of Pathology, Marienkrankenhaus, Hamburg, Germany

^6^ Department of Medical Biometry and Epidemiology, University Medical Center Hamburg-Eppendorf, Hamburg, Germany.

*equally contributed, shared first authorship

**equally contributed, shared last authorship

^+^Corresponding author:

Correspondence and requests for materials should be addressed to J.S. (e-mail: j.schaedler@uke.de) or B.O. (e-mail: b.ondruschka@uke.de).

Supplemental Table 1: Case numbers of the evaluation methods.

|  |  | Total  (n=735) | COVID-19 death  (n=618) | Non-COVID-19 death  (n=47) | Unclear death  (n=70) |
| --- | --- | --- | --- | --- | --- |
| medical records a | | 264 (35.9%) | 214 (34.6%) | 5 (10.6%) | 45 (64.3%) |
|  |  |  |  |  |  |
| pmCT _b_ | | 411 (55.9%) | 353 (57.2%) | 34 (72.3%) | 24 (34.3%) |
|  | isolated _a_ | 157 (21.4%) | 127 (20.6%) | 11 (23.4%) | 19 (27.1%) |
|  | and usMIA _a_ | 20 (2.8%) | 14 (2.3%) | 3 (6.4%) | 3 (4.3%) |
|  | and autopsy _a_ | 224 (30.5%) | 204 (33.0%) | 18 (38.3%) | 2 (2.9%) |
|  | and usMIA and autopsy _a,c_ | 10 (1.4%) | 8 (1.3%) | 2 (4.3%) | - |
|  |  |  |  |  |  |
| usMIA _b_ | | 41 (5.6%) | 31 (5.0%) | 6 (12.8%) | 4 (5.7%) |
|  | isolated _a_ a | 11 (1.5%) | 9 (1.5%) | 1 (2.1%) | 1 (1.4%) |
|  |  |  |  |  |  |
| autopsy _b,c_ | | 283 (38.5%) | 254 (41.1%) | 27 (57.4%) | 2 (2.9%)- |
|  | isolated _b,c_ | 49 (6.7%) | 42 (6.8%) | 7 (14.9%) | - |

This table displays case numbers of the different evaluation methods. Inclusion of one patient in various categories is possible, if two evaluation methods or more were done. Abbreviations: pmCT – postmortem computer tomography, usMIA – ultrasound guided minimal invasive autopsy; _a_ number (%) _b_ multiple inclusion of one patient in the various categories possible, _c_ of all cases 30 autopsies were performed at the Marien-Hospital Hamburg.

Supplemental Table 2: Results of the multiple logistic regression of the COVID-19 death group (I),
and results of the multiple logistic regression in the CA collective of the COVID-19 death group (II).

|  |  | comparison | OR | 95% CI | p-value |
| --- | --- | --- | --- | --- | --- |
| I | Intercept | - | - | - | 0.027 (*) |
|  | Sex | male vs. female | 1.30 | 0.70 – 2.40 | 0.410 |
|  | Age | - | 1.00 | 0.97 – 1.03 | 0.956 |
|  | Place of death | out-patient vs. hospital | 0.28 | 0.15 – 0.52 | <0.001 (***) |
| II | Intercept | - | - | - | 0.165 |
|  | Sex | male vs. female | 0.95 | 0.41 – 2.2 | 0.899 |
|  | Age | - | 1.00 | 0.97 – 1.04 | 0.792 |
|  | Place of death | out-patient vs. hospital | 0.24 | 0.10 – 0.57 | 0.001 (**) |
|  |  |  |  |  |  |
|  | Intercept | - | - | - | <0.001 (***) |
|  | Place of death | out-patient vs. hospital | 0.22 | 0.07 – 0.65 | 0.006 (*) |
|  | Neurological | no vs. yes | 0.20 | 0.06 – 0.73 | 0.015 (*) |
|  |  |  |  |  |  |
|  | Intercept | - | - | - | <0.001 (***) |
|  | Place of death | out-patient vs. hospital | 0.23 | 0.08 – 0.67 | 0.007 (*) |

This table shows in I the results of the multiple logistic regression in the whole collective (event = COVID-19 death) and in II the results of the multiple logistic regression in the CA collective (event = COVID-19 death; in the 5^th^ to 8^th^ line). The results of the multiple logistic regression extended for pre-existing medical conditions (individual) and BMI (event = COVID-19 death) after backward selection are listed in the 9^th^ to 11^th^ and 12^th^ to 13^th^ line. Abbreviations: CA – conventional autopsy.

Supplemental Table 3: Tissue list conventional autopsy.

|  | Tissue | Localisation |
| --- | --- | --- |
| heart/lung-organ block | |  |
|  | lung | UL left subpleural |
|  |  | LL left subpleural |
|  |  | UL right subpleural |
|  |  | LL right subpleural |
|  |  | UL left central |
|  |  | LL left central |
|  |  | UL right central |
|  |  | LL right central |
|  | main bronchus | right |
|  | heart | myocardial front wall |
|  |  | posterior wall |
|  |  | septum |
|  |  | right chamber |
|  |  | right coronay artery |
|  |  | left coroany artery |
|  |  |  |
| sceletal system/soft tissues | |  |
|  | vessels lower limbs | vena saphena right |
|  | bone marrow and soinal cord | part of the vertebral bodies |
|  | muscle | musculus vastus lateralis right |
|  |  | musculus psoas right |
|  | skin | abdomen |
|  |  | head |
|  | soft tissue | abdomen |
|  |  |  |
| neck-organ block | |  |
|  | thyroid gland |  |
|  | throat | mucosa around the hyoid bone |
|  | larynx | mucosa |
|  | N. vagus |  |
|  | trachea middle third |  |
|  | vessels | A. carotis communis right |
|  | lymph nodes | cervical |
|  |  | tracheal bifurcation |
|  |  |  |
| abdomen-organ block | |  |
|  | liver | right lobe |
|  | pancreas |  |
|  | adrenal gland | right side |
|  | spleen |  |
|  | mucosa of the gastrointestinal tract | esophagus |
|  |  |  |
| urogenital-organ block | |  |
|  | kidney | right |
|  |  | left |
|  | prostate |  |
|  | uterus | cervix/corpus |
|  | adnexes/ testicles | right |
|  | vessels | aortic wall |
|  |  | vena cava inferior |
|  | lymph nodes | Inguinal |
|  |  |  |
| colon-organ block | |  |
|  | mucosa of the gastrointestinal tract | duodenum |
|  |  | colon |
|  |  |  |
| additional vessels |  |  |
|  | vessels | peripheral pulmonary arteries |
|  |  | aorta abdominalis |
|  |  | arteria mesenterica superior |
|  |  | vena jugularis |
|  |  | vena cava inferior |
|  |  |  |
| bodily fluids |  |  |
|  | venous blood |  |
|  | urine |  |
|  | intestinal content |  |
|  | vitreous humor |  |
|  | aqueous humor |  |
|  | bile fluid |  |

This table shows the standardized and extended tissue sampling at conventional autopsy, which was performed in case of a short postmortem interval (<72 h). Then, samples were fixed in buffered 4% formaldehyde or were made accessible for further laboratory examination methods by cryopreservation. Abbreviations: UL – upper lobe, LL – lower lobe.

Supplemental Table 4: Tissue list of the ultrasound-guided minimal invasive autopsy.

|  | Tissue | Localisation |
| --- | --- | --- |
| heart/lung-organ block | |  |
|  | lung | UL left |
|  |  | LL left |
|  |  | UL right |
|  |  | LL right |
|  | heart | septum |
|  |  |  |
| abdomen-organ block | |  |
|  | liver | right |
|  | spleen |  |
|  |  |  |
| urogenital-organ block | |  |
|  | kidney | right |
|  |  | left |
|  | prostate |  |
|  | uterus | corpus |
|  |  |  |

This table shows the standardized tissue sampling at ultrasound-guided minimal invasive autopsy, which was performed in case of a short postmortem interval (<72 h). After biopsy, samples were fixed in buffered 4% formaldehyde or were made accessible for further laboratory examination methods by cryopreservation. A sterile 14G (gauge) needle by SOMATEX^®^ (Biopsie Handy, Berlin, Germany) were used for puncture. Abbreviations: UL – upper lobe, LL – lower lobe.
